# The yield of SNP microarray analysis for fetal ultrasound cardiac abnormalities

**DOI:** 10.1186/s12884-024-06428-9

**Published:** 2024-04-05

**Authors:** Fenglei Ye, Xiayuan Xu, Yi Wang, Lifang Chen, Qunda Shan, Qijing Wang, Fan Jin

**Affiliations:** 1 Department of Reproductive Endocrinology, Key Laboratory of Reproductive Genetics of National Ministry of Education, Women’s Hospital, School of Medicine, Zhejiang University, 1 Xueshi Road, Hangzhou, 310000 China; 2 Department of Obstetrics, Lishui Maternal and Child Health Hospital, Lishui, 323000 China; 3 Department of Laboratory, Jinhua Maternal and Child Health Hospital, Jinhua, 321000 China; 4 Department of Gynecology, Women’s Hospital, School of Medicine, Zhejiang University, Hangzhou, 310000 China; 5 Department of Prenatal Diagnosis Center, Lishui Maternal and Child Health Hospital, Lishui, 323000 China

**Keywords:** Single nucleotide polymorphism microarray, Congenital heart disease, Copy number variation, Prenatal diagnosis

## Abstract

**Background:**

Chromosomal microarray analysis (CMA) has emerged as a critical instrument in prenatal diagnostic procedures, notably in assessing congenital heart diseases (CHD). Nonetheless, current research focuses solely on CHD, overlooking the necessity for thorough comparative investigations encompassing fetuses with varied structural abnormalities or those without apparent structural anomalies.

**Objective:**

This study sought to assess the relation of single nucleotide polymorphism-based chromosomal microarray analysis (SNP-based CMA) in identifying the underlying causes of fetal cardiac ultrasound abnormalities.

**Methods:**

A total of 2092 pregnant women who underwent prenatal diagnosis from 2017 to 2022 were included in the study and divided into four groups based on the presence of ultrasound structural abnormalities and the specific type of abnormality. The results of the SNP-Array test conducted on amniotic fluid samples from these groups were analyzed.

**Results:**

Findings from the study revealed that the non-isolated CHD group exhibited the highest incidence of aneuploidy, overall chromosomal abnormalities, and trisomy 18, demonstrating statistically significant differences from the other groups (*p* < 0.001). Regarding the distribution frequency of copy number variation (CNV) segment size, no statistically significant distinctions were observed between the isolated CHD group and the non-isolated CHD group (*p* > 0.05). The occurrence rates of 22q11.2 and 15q11.2 were also not statistically different between the isolated CHD group and the non-isolated congenital heart defect group (*p* > 0.05).

**Conclusion:**

SNP-based CMA enhances the capacity to detect abnormal CNVs in CHD fetuses, offering valuable insights for diagnosing chromosomal etiology and facilitating genetic counseling. This research contributes to the broader understanding of the utility of SNP-based CMA in the context of fetal cardiac ultrasound abnormalities.

**Supplementary Information:**

The online version contains supplementary material available at 10.1186/s12884-024-06428-9.

## Introduction

Congenital heart disease (CHD) is the most common congenital disability, affecting approximately 1% of all births [1]. The prevalence of CHD has continued to increase globally since 2009 [2]. CHD is defined as abnormal development of the cardiovascular system and is characterized by one or more structural heart defects at birth. Although the survival rates of CHD patients have improved due to pediatric cardiovascular surgery and cardiac interventional catheterization, CHD remains the leading cause of death from a congenital anomaly within the first four years of life [3]. Consequently, preconception counseling regarding prenatal screening and the risk of recurrence is urgently needed for CHD patients of reproductive age and women who have previously conceived CHD fetuses.

CHD pathogenesis is known to be multifactorial, with genetic factors playing a significant role in its etiology. A recent study has found that approximately 40% of CHD cases are caused by genetic factors, 5% by environmental variables, and the remaining 55% by unknown factors [4]. Chromosomal abnormalities, copy number variants (CNVs), and single gene disorders comprise most genetic contributions to CHD. Initial studies have shown that trisomy 21, trisomy 18, trisomy 13, and monosomy X are the most common chromosomal aneuploidies in infants with CHD [5]. With the evolution of detection platforms in recent years, there is evidence that genome-wide rare CNVs significantly contribute to CHD susceptibility. CNVs are defined as the gain or loss of genomic material that is 1 kb or larger between individuals of the same species. CNVs can present deletions, duplications, insertions, inversions, and translocations. Robust studies have shown that 22q11.2 deletion syndrome (Del22q11) is estimated to exhibit a prevalence of 50% in interrupted aortic arch type B, 33% in truncus arteriosus, 15% in tetralogy of Fallot (TOF), and 5–10% in ventricular septal defect (VSD) [6, 7]. The deletion or haploinsufficiency of the T-Box transcription factor TBX1 within the 22q11.2 region is closely associated with the cardio-pharyngeal phenotype. Recently, emerging genomic hotspots including 1q21.1, 2q13, 8p23.1, 11q24, 15q11.2, 16p11.2, and 22q11.2 are enriched in CHD cohorts [8]. However, the reduced penetrance and variable phenotype expressivity of some likely pathogenic CNVs have made the prenatal diagnosis of CHD challenging.

Single nucleotide polymorphism array (SNP-Array) is an effective method for detecting CNVs in the genome with high sensitivity for submicroscopic abnormalities. In previous studies, SNP arrays have been conducted to investigate the etiology of CHD [9, 10]. However, the correlation of the detected pathogenic CNVs for CHD differed slightly due to previous studies’ varied cohorts and control settings. Therefore, additional studies are required to emphasize the value of CNV screening as a tool for prenatal CHD diagnosis. In this study, we analyzed 2092 fetuses, aiming to demonstrate the significance of SNP-based chromosomal microarray analysis in investigating the etiology of CHD fetuses.

## Materials and methods

### Participants

**Fig. 1:**
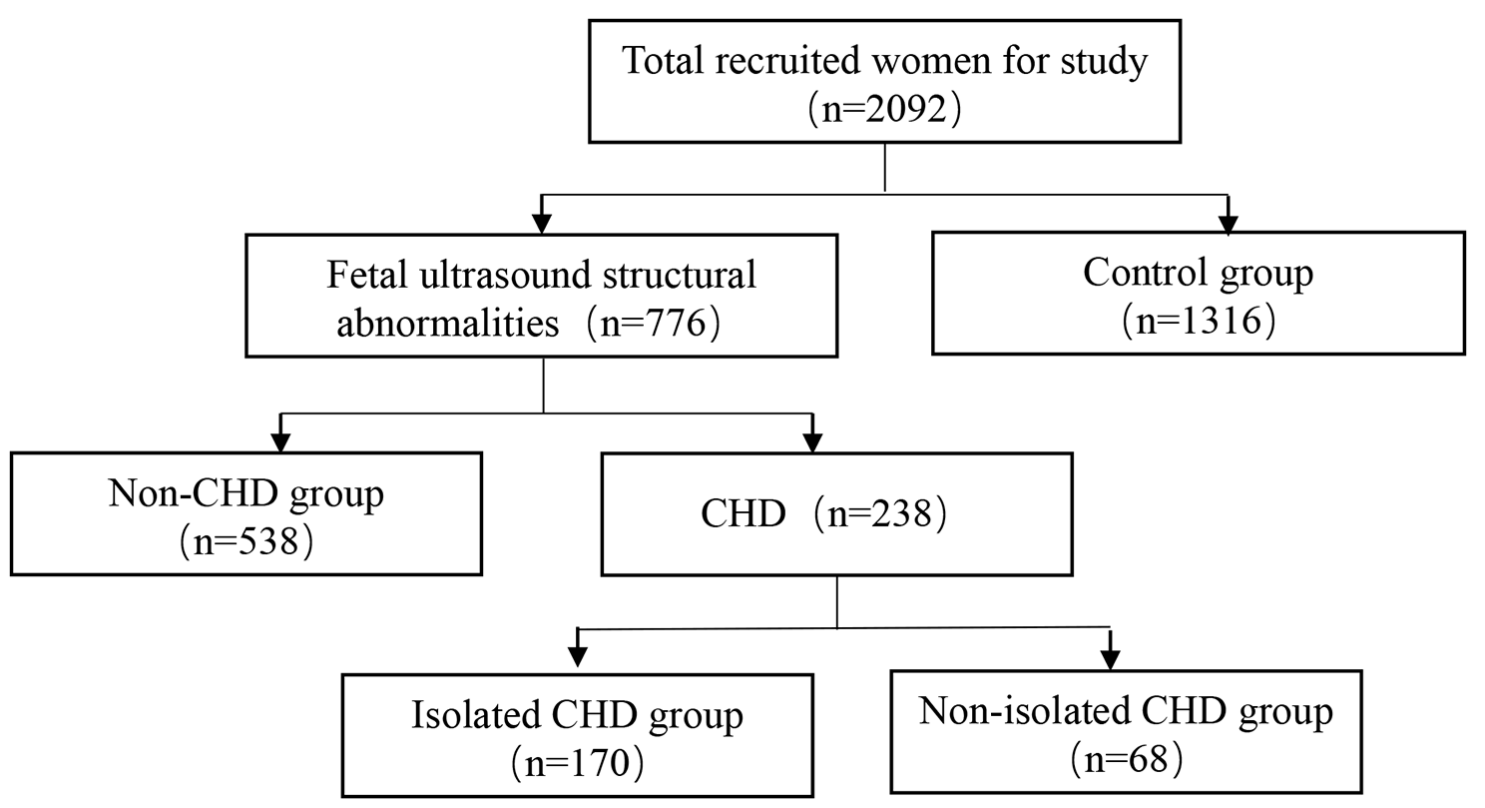
Flowchart for study inclusion describing how the patient cohort was derived

A total of 2092 pregnant women who underwent prenatal diagnosis at the prenatal diagnosis center of Lishui Maternal and Child Health Hospital and Jinhua Maternal and Child Health Hospital from January 2017 to December 2022 were included in the study. Based on prenatal ultrasound examination indicating the presence of fetal structural abnormalities and types of abnormalities, the participants were categorized into four groups: isolated CHD group (170 cases with only fetal heart abnormalities), non-isolated CHD group (68 cases with both fetal heart abnormalities and extracardiac abnormalities, including soft markers), non-CHD group (538 cases with other fetal structural abnormalities, including soft markers, but without fetal heart abnormalities), and control group (1316 cases with no fetal structural abnormalities detected by ultrasound) (Fig. 1). Amniotic fluid from each group underwent chromosomal karyotyping and SNP-Array testing, followed by retrospective analysis.

The inclusion criteria for the control group were as follows: singleton pregnancy that is naturally conceived, gestation age range from 16 to 32 weeks, and the presence of any one or more of the following conditions: (1) high risk of maternal serum biochemical screening or non-invasive prenatal testing (NIPT) positive results; (2) adverse pregnancy history ; (3) maternal age of 35 years or older; (4) Oligohydramnios, exposure to teratogenic substances first trimester and mental disorders. Exclusion criteria include fetal growth restriction, consanguineous marriage, family history of hereditary disease, and pregnant women who received allogeneic blood transfusion, transplantation surgery, or immunotherapy within one year. All pregnant women signed an informed consent form, and this study was approved by the ethics committee of Lishui Maternal and Child Health Hospital and Jinhua Maternal and Child Health Hospital, which serve as tertiary referral centers for prenatal diagnosis.

### Methods

#### Ultrasound examination

Fetal nuchal translucency (NT) examinations were conducted at 11–14 weeks, and structural screening was performed at 16–32 weeks. If fetal structural abnormalities were identified before 18 weeks gestation, a follow-up ultrasound examination for confirmation was scheduled at 22–26 weeks. The Voluson E8 ultrasound diagnostic instrument (GE company) was utilized, equipped with two-dimensional (2D) probes of C2-9 (frequency 2 ∽ 9 MHz) and C1-5 (frequency 1 ∽ 5 MHz), as well as the fetal cardiac mode was used to examine the fetal heart. The diagnostic protocols adhered to the guidelines outlined by the International Society of Ultrasound in Obstetrics and Gynecology guidelines [11, 12] in various planes. Fetal heart defects were categorized according to the International Pediatric and Congenital Heart Disease Code (IPCCC) and International Classification of Diseases 11th Revision (ICD-11) [13]. At least two ultrasound physicians with prenatal diagnosis qualifications were recruited to perform the assessments.

#### Amniocentesis and SNP-array testing

Amniocentesis was performed at 16–28 weeks’ gestation. Under real-time ultrasound guidance, biopsy needles (PCN21/15 or 22/20, GALLINIS.R.L, Italy) were used to extract 30 ml of amniotic fluid, with 20 ml allocated for chromosomal karyotype analysis. The remaining 10 ml underwent DNA extraction using the QIAamp DNA kit (Jant Pharmacal Corporation, USA), followed by SNP-array testing using the Affymetrix CytoScan 750 K chip provided by Affymetrix, USA. All samples were collected from amniotic fluid.

### SNP-array result interpretation

The ChAs software (Thermo Fisher, Affymetrix, USA) was utilized to interpret the detection results based on multiple databases, including the Database of Genomic Variants (http://projects.tcag.ca/variation/), DECIPHER (http://decipher.sanger.ac.uk/); ClinVar (https://www.ncbi.nlm.nih.gov/clinvar/); and Online Mendelian Inheritance in Man (http://www.ncbi.nlm.nih.gov/omim). Cases with copy number gains or losses involving the entire chromosome were defined as aneuploid, while deletions greater than 100 kb and duplications greater than 200 kb were considered CNV. The qualitative assessment of CNV was classified according to the American College of Medical Genetics and Genomics (ACMG) guidelines, including pathogenic, likely pathogenic, and variants of uncertain significance [14]. The occurrence rates and distribution segments of aneuploid, pathogenic, likely pathogenic, and variants of uncertain significance CNVs among the four groups were compared to provide information for the SNP-array detection technology in the etiology of fetal CHD.

### Statistical analysis

The data was processed using SPSS version 25.0 (IBM Corporation, Armonk, NY, USA). Normally, the assumption was assessed for continuous variables, and for those meeting this criterion, one-way analysis of variance (ANOVA) followed by post hoc pairwise comparisons were applied. Nonparametric multiple sample tests, specifically the Kruskal-Wallis test, were conducted in instances of non-normally distributed or unequal variances among continuous variables, followed by post hoc pairwise comparisons. Categorical variables were compared among multiple groups using the chi-square test, with statistical significance set at *p* < 0.05.

## Results

### Demographical characteristic

The age and gestational weeks of the four groups of pregnant women they have exhibited a non-normal distribution. In the isolated CHD group, maternal ages ranged from 17 to 44 years, and the gestational weeks ranged from 16 to 28 weeks. For the non-isolated congenital heart group, the maternal ages ranged from 18 to 42 years, and gestational weeks ranged from 16 to 31 weeks. The non-CHD group displayed maternal ages ranging from 19 to 46 years and gestational weeks from 16 to 32 weeks. Similarly, the control group exhibited maternal ages ranging from 18 to 46 years, and the gestational weeks ranged from 17 to 32 weeks. Table 1 displays the demographic and diagnostic information of the four groups. No statistically significant differences were observed in age and ultrasound-diagnosed gestational weeks among the four groups ( *p* = 0.610, 0.151). Supplementary Table 1 summarizes CHD types and the proportion of pathogenic findings for fetuses with CHD across different groups. Additionally, Supplementary Table 2 details the soft marker types and the proportion of pathogenic findings for fetuses with non-isolated CHD and non-CHD. The composition of the control group is outlined in Supplementary Table 3.

**Table 1:** Maternal age and gestational weeks of four groups (Mean ± SD)

	Total	Age	gestational week
Isolated CHD group	170	31.12 ± 5.01	21.69 ± 2.81
Non-isolated CHD group	68	31.09 ± 5.66	21.94 ± 3.48
Non-CHD group	538	31.03 ± 5.51	21.47 ± 3.29
Control group	1316	31.60 ± 6.91	21.24 ± 2.44
*P*-value		0.610	0.151

### Incidence of aneuploidy and CNV segments with various properties

Table 2 shows the incidence of aneuploidy, pathogenic CNVs, likely pathogenic CNVs, and variants of uncertain significance CNVs in the four groups. Concerning the incidence of aneuploidy, the non-isolated CHD group displays the highest rate, with significant statistical differences observed when compared to the isolated CHD group (27.9% vs. 3.5%, *p* < 0.001), the non-CHD group (27.9% vs. 8.6%, *p* < 0.001), and the control group (27.9% vs. 5.9%, *p* < 0.001). In terms of the incidence of pathogenic CNVs and 22q11.2, no statistically significant differences were found between the isolated CHD group and the non-isolated CHD group (8.2% vs. 14.7%, *p* = 0.134) (2.9% vs. 4.4%, *p* = 0.865). The incidence of overall chromosomal abnormalities in the non-isolated CHD group was highest, with significant statistical distinctions compared to the isolated CHD group (42.6% vs. 14.1%, *p* < 0.001), the non-CHD group (42.6% vs. 14.9%, *p* < 0.001), and the control group (42.6% vs. 9.4%, *p* < 0.001). Regarding the incidence of variants of uncertain significance CNVs, no statistically significant differences existed between the isolated CHD group and the non-isolated CHD group(8.2% vs. 8.8%, *p* = 0.883).

**Table 2:** Incidence of aneuploidy, pathogenic, likely pathogenic, and variants of uncertain significance CNVs in four groups of pregnant women (%)

	n	aneuploidy	Pathogenic CNVs			Likely pathogenic CNVs	Total known pathogenic and likely pathogenic findings	variants of uncertain significance CNVs
			22q11.2	Other	Total			
Isolated CHD group	170	6(3.5)	5(2.9)	9(5.3)	14(8.2)	0(0)	20(11.8)	14(8.2)
Non-isolated CHD group	68	19(27.9)	3(4.4)	7(10.3)	10(14.7)	0(0)	29(42.6)	6(8.8)
Non-CHD group	538	46(8.6)	2(0.4)	28(5.2)	30(5.6)	2(0.4)	78(14.5)	28(5.2)
Control group	1316	78(5.9)	5(0.4)	37(2.8)	42(3.2)	3(0.4)	123(9.3)	41(3.1)
*P*-value		< 0.001	< 0.001	0.005	< 0.001	-	< 0.001	0.031

### Distribution of aneuploidy

Table 3 lists the distribution of aneuploidy. Regarding the incidence of trisomy 21, statistically significant differences were observed only between the non-CHD group and the control group (*p* = 0.003), with no significant differences noted among the other groups. For trisomy 18, the non-isolated CHD group exhibited the highest incidence rate, showing significant statistical significance when compared to the isolated CHD group (16.2% vs. 1.2%, *p* < 0.001), the non-CHD group (16.2% vs. 1.5%, *p* < 0.001), and the control group (16.2% vs. 0.5%, *p* < 0.001). No statistical differences were found between the isolated CHD, non-CHD, or control groups. Regarding trisomy 13, a statistically significant difference was observed between the non-isolated CHD and non-CHD groups (4.4% vs. 0.4%, *p* = 0.008).

**Table 3:** The distribution of aneuploidy in four groups of pregnant women (%)

	n	Trisomy 21	Trisomy 18	Trisomy 13	Other aneuploidies
Isolated CHD group	170	4(2.4)	2(1.2)	0	0
Non-isolated CHD group	68	4(5.9)	11(16.2)	3(4.4)	1(1.5)
Non-CHD group	538	28(5.2)	8(1.5)	2(0.4)	8(1.5)
Control group	1316	33(2.5)	6(0.5)	0	39(3.0)
*P*-value		0.021	< 0.001	-	-

### Distribution frequency of CNV fragment sizes

We analyzed and compared CNV fragment sizes across four groups, encompassing pathogenic CNVs, likely pathogenic CNVs, and variants of uncertain significance CNVs, as detailed in Table 4. Regarding the distribution frequency of CNVs < 1 Mb, statistically significant differences were solely noted between the non-CHD group and the control group (*p* = 0.002). No statistically significant difference emerged between the isolated CHD group and the non-isolated CHD group (4.7% vs. 5.9%, *p* = 0.963). For CNVs with a distribution frequency of 1-4 Mb, statistically significant differences were found between the isolated CHD group, the non-isolated CHD, and the control group (7.1% vs. 3.3%, *p* = 0.014; 9.1% vs. 3.3%, *p* = 0.031). However, no statistically significant difference was found between the isolated CHD group and the non-isolated CHD group (7.1% vs. 9.1, *p* = 0.598). Concerning CNVs with a distribution frequency ≥ 5 Mb, the non-isolated CHD group exhibited the highest frequency. However, no statistically significant difference emerged between the non-isolated CHD group and the isolated CHD group (8.8% vs. 4.7%, *p* = 0.360). Nonetheless, statistically significant differences were evident between the non-isolated CHD group and the non-CHD group (*p* = 0.003), and the control group (*p* < 0.001).

**Table 4:** Compares the distribution frequencies of CNV fragment sizes among four groups of pregnant women (%)

	n	< 1 Mb	1-4 Mb	≥ 5 Mb
Isolated CHD group	170	8(4.7)	12(7.1)	8(4.7)
Non-isolated CHD group	68	4(5.9)	6(8.8)	6(8.8)
Non-CHD group	538	25(4.6)	25(4.6)	10(1.9)
Control group	1316	27(2.1)	43(3.3)	16(1.2)
*P*-value		0.008	0.030	< 0.001

### The distribution profiles of pathogenic CNVs in the four groups

As seen in Table 5, the isolated CHD group comprised two large segmental duplications: partial trisomy 4p and Xq28 duplication syndrome, four cases of large segmental deletions (7.2 ∽ 12.3 Mb), one case of < 4 Mb duplication (Cat eye syndrome), and seven cases of < 4 Mb deletion, of which four 22q11.2 microdeletion syndrome, one 12q14 microdeletion syndrome, Del(19p13.2), and 15q11.2 BP1-BP2 microdeletion. The non-isolated CHD group comprised three cases of large segment duplications (13.37 ∽ 47.7 Mb), two cases of large segment deletions (10.1 ∽ 14.9 Mb), and five cases of deletions < 4 Mb. Among these are one 15q11.2 microdeletion and three 22q11.2 microdeletion syndromes. The non-CHD group had three cases of large segment duplications (12.7 ∽ 22.6 Mb), six cases of large segment deletions (6.3 ∽ 19.1 Mb), sixteen cases of deletions, and five cases of duplication < 4 Mb. Among these, known microduplications/microdeletions as 15q11.2 BP1-BP2 microdeletion, 16p11.2 microdeletion/microduplication syndrome, Potocki-Lupski syndrome, 22q11.2 deletion/duplication syndrome, and Leri-Weill dyschondrostosis (LWD)-SHOX deletion. The control group included three large segment duplications (8.1 ∽ 22.5 Mb), twelve large segment deletions (5.4 ∽ 25.7 Mb), and twenty-seven cases of microdeletions/microduplications. Among these, known CNVs included 1q21.1 recurrent microduplication, 7q11.23 duplication syndrome, 15q11.2 BP1-BP2 microdeletion, 16p11.2 microduplication syndrome, 22q11.2 deletion/duplication syndrome, Cat eye syndrome, and others.

**Table 5:** Distribution of pathogenic CNV in the four groups

Isolated CHD group	Non-isolated CHD group	Non-CHD group	Control group	Correspondence syndrome
Del(1q43q44) (9.8 Mb)				1q43q44 deletion syndrome
			Del(1q21.1q21.2)(5.4 Mb)	1q21.1 recurrent microdeletion
			Dup(1q21.1q21.2)(1.8 Mb)	1q21.1 recurrent microduplication
			Dup(1q21.1q21.2)(2.1 Mb)	1q21.1 recurrent microduplication
			Del(2q14.2q22.3)(25.0 Mb)	
	Del(2p25.3)(2.6 Mb)		Del(2p25.1p24.2)(5.8 Mb)	
	Dup(2p25.3p21)(41.1 Mb)			
		Del(2q37.1q37.3)(9.3 Mb)		
			Del(3p26.3p25.3)(11.5 Mb)	
Dup(4p16.3p14)(39.7 Mb)				Partial trisomy 4p
		Del(4p16.3p15.31)(19.1 Mb)	Del(4p16.3p15.32)(16.3 Mb)	Wolf-Hirschhorn syndrome
		Del(4p16.3p15.33)(13.5 Mb)		Wolf-Hirschhorn syndrome
			Del(5p15.33p14.1)(25.7 Mb)	
		Dup(5q35.2q35.3)(1.9 Mb)	Del(5q35.3)(3.1 Mb)	
			Del(5q35.2q35.3)(2.1 Mb)	
		Del(6q26q27)(8.4 Mb)	Del(6p24.3)(526.6Kb)	
			Dup(7q11.23)(1.4 Mb)	7q11.23 duplication syndrome
		Del(7q11.23)(1.5 Mb)		
	Dup(8q22.1q24.3)(47.7 Mb)			
			Del(8p23.3p23.1)(10.2 Mb)	
			Del(8p23.3p23.1)(8.2 Mb)	
			Dup(9p24.3p21.3)(22.5 Mb)	
	Del(10p15.3p14)(10.1 Mb)			
			Del(10q23.31)(1.0 Mb)	
			Dup(10q25.3q26.3)(19.0 Mb)	
Del(10q26.13q26.3)(10.8 Mb)				10q26 deletion syndrome
		Dup(11p12p11.12)(12.7 Mb)		
			Del(11p14.3p13)(10.4 Mb)	
Del(11q24.2q25)(7.2 Mb)				
Del(11q24.1q25)(12.3 Mb)				
Del(12q14.3q15)(3.14 Mb)				12q14 microdeletion syndrome
		Dup(12p13.33p12.1)(22.6 Mb)		
			Del(13q13.3q14.3)(14.5 Mb)	
	Dup(14q22.1q23.3)(13.37 Mb)			
Del(15q11.2)(512.3Kb)	Del15q11.2)(507.0Kb)	Del(15q11.2)(855.3Kb)	Del(15q11.2)(855.4Kb)	15q11.2 BP1-BP2 microdeletion
		Del(15q11.2)(855.3Kb)		15q11.2 BP1-BP2 microdeletion
		Del(15q11.2)(512.4Kb)		15q11.2 BP1-BP2 microdeletion
		Del(15q11.2)(311.8Kb)		15q11.2 BP1-BP2 microdeletion
		Del(15q11.2)(855.3Kb)		15q11.2 BP1-BP2 microdeletion
		Del(15q11.2)(507.0Kb)		15q11.2 BP1-BP2 microdeletion
		Del(16p11.2)(749.9Kb)		16p11.2 microdeletion syndrome
		Del(16p11.2)(309.5Kb)		16p11.2 microdeletion syndrome
		Del(16p11.2)(622.7Kb)		16p11.2 microdeletion syndrome
		Del(16p11.2)(301.9Kb)		16p11.2 microdeletion syndrome
		Dup(16P11.2)(226.7Kb)	Dup(16p11.2)(585.1Kb)	16p11.2 microduplication syndrome
		Dup(16P11.2)(598.7Kb)	Dup(16p11.2)(585.2Kb)	16p11.2 microduplication syndrome
		Del(16p13.12p12.3)(2.1 Mb)		
		Dup(17p11.2)(3.7 Mb)		Potocki-Lupski syndrome
	Del(18p11.32p11.21)(14.9 Mb)	Del(18p11.32p11.21)(14.9 Mb)		Partial monosomy 18p
			Del(18q21.31q23)(22.2 Mb)	
Del(19p13.2)(2.1 M)				
		Dup(21q21.2q22.13)(14.8 Mb)	Dup(21q22.2q22.3)(8.1 Mb)	
		Del(21q22.3)(1.6 Mb)		
Del(22q11.21)(3.1 Mb)	Del(22q11.21)(2.8 Mb)	Del(22q11.1q11.21)(3.8 Mb)	Del(22q11.21)(744.1Kb)	22q11.2 deletion syndrome
Del(22q11.21)(3.1 Mb)	Del(22q11.21)(3.1 Mb)		Del(22q11.21)(2.8 Mb)	22q11.2 deletion syndrome
Del(22q11.21q11.22)(1.1 Mb)	Del(22q11.21)(2.9 Mb)		Del(22q11.21)(3.2 Mb)	22q11.2 deletion syndrome
Del(22q11.21)(3.2 Mb)				22q11.2 deletion syndrome
		Dup(22q11.21)(2.8 Mb)	Dup(22q11.21)(2.8 Mb)	22q11.2 duplication syndrome
Dup(22q11.1q11.21)(1.6 Mb)			Dup(22q11.1q11.21)(2.1 Mb)	Cat eye syndrome
			Del(22q12.1)(213.4Kb)	
			Del(22q13.32q13.33)(2.5 Mb)	
			Del(Yq11.221q11.23)(10.5 Mb)	AZFb + AZFc deletion
			Del(Yq11.223q11.23)(3.5 Mb)	AZFc deletion
		Del(Xp21.1)(272.1Kb)	Del(Xp21.1)(271.3Kb)	
			Del(Xp22.31)(1.2 Mb)	Steroid sulphatase deficiency(STS)
			Del(Xp22.31)(1.6 Mb)	Steroid sulphatase deficiency(STS)
			Del(Xp22.31)(1.6 Mb)	Steroid sulphatase deficiency(STS)
			Del(Xp22.31)(1.6 Mb)	Steroid sulphatase deficiency(STS)
			Del(Xp22.31)(1.7 Mb)	Steroid sulphatase deficiency(STS)
			Del(Xp22.31)(1.7 Mb)	Steroid sulphatase deficiency(STS)
			Del(Xp22.31)(1.7 Mb)	Steroid sulphatase deficiency(STS)
		Del(XP22.33)(3.9 Mb)	Del(Xp22.33)(742.6Kb)	Leri-Weill dyschondrostosis (LWD) - SHOX deletion
		Del(Xp22.33p22.31)(6.3 Mb)		Leri-Weill dyschondrostosis (LWD) - SHOX deletion
Dup(Xq27.2q28)(13.1 Mb)				Xq28 duplication syndrome

### Distribution profile of likely pathogenic CNVs in the four groups

Table 6 shows that in the non-CHD group, there was one case each of duplication and deletion of CNV. In the control group, there was one case of a large segment duplication and two cases of microdeletions.

**Table 6:** Distribution of likely pathogenic CNV in the four groups

Isolated CHD group	Non-isolated CHD group	Non-CHD group	Control group
			Dup(4p16.3p16.1)(10.7 Mb)
		Dup(11p15.5p15.4)(5.1 Mb)	
		Del(16p12.2)(600.7Kb)	Del(16p12.2)(600.6Kb)
			Del(20p13)(981.6Kb)

### Distribution variants of uncertain significance CNVs in four groups

The data in Table 7 provides a clear overview of the distribution of microdeletions and microduplications among the specified groups. In the isolated CHD group, there were six microdeletions and eight microduplications. In contrast, all six cases in the non-isolated CHD group were duplications, including one case of a large segment duplication. The non-CHD group exhibited twenty-one cases of microduplications and seven cases of microdeletions. Finally, the control group showed 23 cases of microduplications and 18 cases of microdeletions.

**Table 7:** Distribution variants of uncertain significance CNVs in the four groups

Isolated CHD group	Non-isolated CHD group	Non-CHD group	Control group
	Dup(1q21.1)(576.0Kb)	Dup(1q21.1)(385.9Kb)	
		Del(1q24.3q25.1)(2.3 Mb)	
			Dup(2p12)(1.4 Mb)
			Dup(2p16.1p15)(1.6 Mb)
Del(2p21)(151.7Kb)			
			Dup(2p25.3)(1.0 Mb)
Del(2q13)(106.4Kb)		Del(2q13)(103.5Kb)	Del(2q13)(871.1Kb)
Del(2q13)(482.1Kb)			Del(2q13)(482.1Kb)
			Del(2q13)(103.5Kb)
			Del(2q13)(1.7 Mb)
			Del(2q13)(1.7 Mb)
		Dup(2q12.3q13)(2.4 Mb)	
	Dup(3p14.2)(1.6 Mb)		
			Dup(3p24.1p23)(4.0 Mb)
			Del(3p25.3)(128.7Kb)
	Dup(3q23q24)(5.18 Mb)		
			Dup(3q25.32)(568.0Kb)
			Dup(4p16.1)(1.9 Mb)
			Del(4p16.2)(1.0 Mb)
			Del(4q34.3)(3.3 Mb)
			Del(4q35.2)(1.3 Mb)
			Dup(5q23.1q23.2)(4.4 Mb)
Dup(5q35.3)(1.6 Mb)			
Del(6p21.31)(1.8 Mb)			
		Dup(6q14.1)(823.2Kb)	Del(6q26)(188.7Kb)
		Dup(6q15)(627.8Kb)	Del(6q26)(241.4Kb)
Dup(6q25.3q26)(1.2 Mb)			
		Dup(7p14.1)(1.3 Mb)	
		Dup(7p21.1)(613.9Kb)	
		Dup(7p21.3)(1.1 Mb)	
			Dup(7p22.3)(789.6Kb)
			Del(7q32.1)(294.0Kb)
			Dup(8p21.3)(2.1 Mb)
			Del(8p23.1)(143.8Kb)
		Dup(8p23.2)(1.3 Mb)	Dup(8p23.3p23.2)(2.7 Mb)
		Dup(8p23.2)(2.2 Mb)	
		Del(8p23.2)(1.1 Mb)	
		Del(8q11.21)(1.7 Mb)	
		Del(9p24.1)(197.9Kb)	
Dup(9p24.3p24.1)(5.3 Mb)			Dup(9p24.3)(686.3Kb)
		Dup(9q31.1)(1.2 Mb)	
			Del(10q21.3)(312.3Kb)
			Del(10q21.3)(1.3 Mb)
Dup(11p14.3p14.1)(4.4 Mb)			
		Del(12q21.32)(1.2 Mb)	
			Dup(13q12.12)(1.4 Mb)
			Del(13q33.1q33.2)(1.8 Mb)
			Del(14q22.1q22.2)(3.1 Mb)
	Dup(15q13.3)(422.4Kb)		Dup(15q13.1q13.2)(1.5 Mb)
		Dup(16p13.11)(1.6 Mb)	Dup(16p13.11) (926.6Kb)
		Dup(16p13.11)(827.3Kb)	Dup(16p13.11) (796.4Kb)
			Dup(16p13.11) (1.2 Mb)
			Dup(16p13.11) (1.6 Mb)
			Dup(16p13.11) (796.4Kb)
Dup(16p13.2p13.13)(486.1Kb)			
	Dup(16p13.3)(1.0 Mb)		
	Dup(16q11.2q12.1)(640.9Kb)	Dup(16q12.1)(480.0Kb)	
Dup(16q22.2)(607.2Kb)			
Del(16q23.1)(260.2Kb)			
			Dup(17p11.2q11.1)(3.7 Mb)
		Dup(17p13.3)(836.1Kb)	
			Del(17p13.3)(624.6Kb)
		Dup(17q22)(1.0 Mb)	
Dup(18p11.32p11.23)(7.8 Mb)			Dup(18p11.32)(1.6 Mb)
		Dup(20q12)(1.4 Mb)	
Dup(21q11.3)(731.9Kb)			
		Del(21q21.1)(1.9 Mb)	
			Dup(21q21.1)(1.2 Mb)
Dup(22q11.23)(1.3 Mb)		Dup(22q11.23)(1.3 Mb)	
		Dup(22q11.23)(1.3 Mb)	
		Dup(Xp22.31)(125.8Kb)	Dup(Xp22.31)(1.6 Mb)
		Dup(Xp22.31)(543.7Kb)	Dup(Xp22.33)(392.7Kb)
		Dup(Xq22.1)(599.2Kb)	

## Discussion

In recent years, the role of identifying chromosomal imbalances through microscopic analysis in the context of congenital heart disease has garnered significant attention [7]. Our study employed fetal ultrasound imaging to categorize individuals into isolated CHD, non-isolated CHD, non-CHD, and control groups. By examining the distribution and occurrence of copy number variations (CNVs) within each group, we aimed to evaluate the feasibility of using SNP array technology to identify the underlying causes of CHD in fetuses. The overall detection rate of abnormal CNVs in isolated CHD and non-isolated CHD groups was 8.2% (14/170) and 14.7% (10/68), respectively. In contrast, the rates in the non-CHD and control groups were 5.6% (30/538) and 3.2% (42/1316), respectively. Notably, related studies have reported a detection rate of 4–20% for chromosomal microarray analysis in CHD [15]. Contrarily, another study revealed a 6% detection rate of chromosomal microarray in cases with a normal karyotype but abnormal ultrasound findings and approximately 1.7% in cases with advanced maternal age, positive chromosomal screening, and other non-ultrasound structural abnormalities [16].

Our study demonstrated a consistent CHD and other ultrasound structural abnormalities detection rate compared to prior research. However, the detection rate was slightly higher in cases without ultrasound structural abnormalities. This difference may be attributed to an increased risk of chromosomal abnormalities in the control group, particularly in individuals of advanced maternal age or those identified as high risk through NIPT. Additionally, variations in the array platform, the resolution of the array, and reporting practices in each clinical laboratory may contribute to these disparities. Furthermore, with new literature and public data sharing, genomic regions definitively associated with the diseases continue to expand. Annual reviews of the same dataset have resulted in an increase in pathogenic cases to 1.8%, while cases of variants of uncertain significance have decreased to 0.9%[17].

A recent meta-analysis of 45 studies revealed that the overall prevalence of chromosomal abnormalities in isolated CHD and non-isolated CHD was 16% and 37%, respectively. Among non-isolated CHD cases, the prevalence of aneuploidy (19%), other CNVs (excluding 22q11) (4%), and trisomy 18 were higher than in isolated CHD cases [18]. Our outcomes demonstrated that the overall prevalence of chromosomal abnormalities in the isolated CHD group and non-isolated CHD group is 11.8% and 42.6%, respectively. Comparative analysis showed that the non-isolated CHD group has the highest prevalence of aneuploidy and overall chromosomal abnormalities, with statistically significant differences compared to the isolated CHD, non-CHD, and control groups. The non-isolated CHD group had the highest prevalence of T18, and there was no statistically significant difference in the prevalence of T21 compared to the isolated CHD group, which is consistent with the meta-analysis. However, there was no statistically significant difference between the non-isolated CHD group and the isolated CHD group (14.7% vs. 8.2%, *p* = 0.134) in the prevalence of pathogenic CNV. Even after excluding 22q11, the non-isolated CHD group still showed no statistically significant difference in pathogenic CNV prevalence compared to the isolated CHD group (10.3% vs. 5.3%, *p* = 0.269), which differs from the meta-analysis. Furthermore, our study also showed that the prevalence of aneuploidy cases and pathogenic CNVs was slightly higher than in the meta-analysis, possibly due to differences in the number of subjects included in each group.

The conventional chromosomal karyotype resolution, typically within the range of 5-10 Mb, has been a standard in genetic analysis. Our research findings, however, reveal no statistically significant difference in the distribution frequency of pathogenic CNVs and variants of uncertain significance CNVs between isolated and non-isolated CHD groups. The significance of CNV segment size is notably contingent upon its genomic location and the inclusion of genes or non-coding regions within its boundaries. Smaller CNV segments may exert localized effects on gene expression, potentially influencing an individual’s phenotype and susceptibility to certain diseases; larger CNV segments can impact multiple genes, thus influencing a spectrum of phenotypes and disease susceptibilities [19]. Some studies suggest that the etiology of isolated CHD is multifactorial, with some cases being attributed to single genes. Non-isolated CHD is associated with various causes, including chromosomal and sub-chromosomal abnormalities, single-gene syndromes, epigenetic factors, and environmental influences [15]. The results of our study show that in the non-CHD group, pathogenic CNVs larger than 10 Mb account for 50% of the cases. These larger segments may be considered partial aneuploidy, and non-CHD with multiple systemic developmental abnormalities are more likely to manifest as chromosomal abnormality syndromes. In contrast, isolated CHD identified by ultrasound is more likely to be caused by smaller chromosomal segments or gene variations.

In the four pathogenic CNV groups, we identified 15 cases involving the 22q11.2 region, including 11 deletions and 4 duplications. The 22q11.2 microdeletion is currently recognized as a syndrome associated with congenital heart defects, with severity ranging from non-survivable to subclinical or even without a CHD phenotype. The main known causative gene for this syndrome is TBX1. The 22q11.2 microduplication syndrome complements the 22q11.2 microdeletion syndrome, sharing common features but exhibiting considerable phenotypic variation, with a CHD occurrence rate of approximately 25% [20, 21]. Furthermore, we observed one case each of 22q11.1q11.21 microduplication in the isolated congenital heart defect group and the control group. This region is critical within the 22q11.21 recurrent region (Cat Eye Syndrome, CES). However, it does not involve the 22q11.2 region associated with DiGeorge syndrome/Velocardiofacial syndrome (DGS/VCFS) [22]. Upon pairwise comparison, we found that the occurrence rate of 22q11.2 abnormalities in the isolated CHD and non-isolated CHD groups did not exhibit statistically significant differences (2.9% vs. 4.4%, *p* = 0.865), consistent with some previous reports [18, 23]. However, the isolated and non-isolated CHD groups had higher occurrence rates than the non-CHD group (*p* = 0.012, *p* = 0.008) and the control group (*p* = 0.001, *p* = 0.001). Our study revealed the presence of 3 cases of 22q11.2 microdeletion and 2 cases with microduplication in the control group despite the absence of ultrasound-detected structural abnormalities. This phenomenon may be associated with the challenge of identifying neurological and psychiatric characteristics during the prenatal stage, coupled with the absence of typical facial features that would conventionally signal the presence of such genomic variations.

Moreover, across the four groups, a total of 9 cases involved the 15q11.2, with occurrence rates as follows: 0.6% (1/170) in the isolated CHD group, 1.5% (1/68) in the non-isolated CHD group, 1.1% (6/538) in the non-CHD group, and 0.1% (1/1316) in the control group. Post-pairwise comparisons revealed no statistically significant differences between the isolated CHD group and the non-isolated CHD group (*p* = 0.522). The nature of the 15q11.2 BP1-BP2 microdeletion has been controversial, underscoring the complexity and ongoing discussions in understanding this specific genomic anomaly. Its prevalence in CMA-tested populations is approximately 0.57-1.27% [24]. Clinical phenotypes are mainly associated with neurodevelopmental disorders, developmental and language delays, and autism spectrum disorders, with a relatively low penetrance of 10–12%[25]. The 15q11.2 BP1-BP2 microdeletion encompasses four highly conserved non-imprinted genes: NIPA1, NIPA2, CYFIP1, and TUBGCP5. There is no established independent connection between this microdeletion and heart morphology. Some studies suggested that the 15q11.2 BP1-BP2 microdeletion has a relative frequency of 3.4% in intellectual disability, 2% in schizophrenia, and 2.1% in epilepsy, with no increased risk of cardiac malformation or autism, making it of limited clinical significance, and it has been suggested to be classified as a “mildly pathogenic factor.” [26]. In 2015, the UK Genomic Medicine Committee even proposed not reporting the 15q11.2 BP1-BP2 microdeletion in prenatal diagnoses [27]. However, a recent study by the Williams team indicated an increased risk of cardiovascular malformation associated with the 15q11.2 BP1-BP2 microdeletion, with cardiovascular malformation being more common but not necessarily severe [25]. In our study, both isolated and non-isolated CHD groups had 15q11.2 microdeletion, and both exhibited ventricular septal defects, which aligns with the findings by Williams et al. In the non-CHD group, five cases of 15q11.2 microdeletion only showed increased nuchal translucency on ultrasound.

In variants of uncertain significance CNVs, small segmental duplications were predominant across the four groups. It was observed that 2q13 was involved in isolated CHD, non-CHD, and control groups, with eight cases of microdeletions and one case of microduplication—the pathogenic nature of 2q13 needs to be better understood. Several studies have indicated that duplications and deletions of 2q13 are risk factors for developmental delay and anomalies. Wolfe’s research found an increased prevalence of attention deficit hyperactivity disorder (ADHD) in individuals with defects associated with the 2q13 locus, with 30% of defect carriers having heart defects. In contrast, no defects were observed in carriers of duplications [28]. Other researchers have noted that with chromosome 2q13 phenotypes, deletions are more enriched in cardiovascular disease, while duplications are associated with craniofacial features [29]. Our study found two cases of 2q13 deletion in individuals with CHD, and both were isolated. Their phenotypes were complete transposition of the great arteries with pulmonary artery stenosis and anomalous origin of the right pulmonary artery with pulmonary artery stenosis, which aligns with the above results. Since 2q13 can also be present in the normal population and given the current lack of large-sample data, further research may be needed to explore the pathogenic genes and phenotypes associated with heart defects.

Additionally, we also observed that the isolated CHD group had a Dup(9p24.3p24.1), the control group had a Dup(9p24.3), the non-isolated CHD group had a Dup(16q11.2q12.1), and the non-CHD group had a Dup(16q12.1), all of which involved partially overlapping regions. The 9p24.3 duplication segment contains genes like DOCK8 and may be associated with autism spectrum disorders, intellectual disabilities/developmental delay, and other conditions. However, whether the 9p24.3p24.1 segment is related to congenital heart disease phenotypes has not been reported in the related literature [30]. All four groups of CNVs involved chromosome 16, but the specific segments affected were different. Chromosome 16 is one of the most enriched chromosomes for segmental duplications, and 16p is one of the more unstable regions in the genome, with over 10% of the 16p euchromatic regions comprising highly complex low-copy repeats [31, 32]. The shared regions affected by CNVs in the non-isolated CHD group and the non-CHD group have rare clinical phenotypes reported in previous studies within the community. It suggests that, apart from genetic factors, other factors, such as environmental influences, may also play a role.

Traditional karyotype analysis is labor-intensive and requires extensive manual preparation. The advent of SNP-Arrays has drastically reduced the cost of analyzing constitutional disorders. Consumer usage and commercial platforms have led to declining array and reagent production costs [33]. Understanding the proper clinical applications of SNP-array is still challenging, and the research community still needs new methods to detect real chromosomal imbalance aberrations and the difficulty in interpreting the pathogenicity of many tiny fragments.

### Strengths and limitation

This research study stands out due to its incorporation of a substantial sample size comprising fetuses with various ultrastructural anomalies and those without such anomalies. The comprehensive analysis comparing congenital heart disease (CHD) with these diverse groups, namely isolated CHD, non-isolated CHD, other structural ultrastructural anomalies, and those without structural ultrastructural anomalies, offers a unique opportunity to discern potential differences and commonalities among them. This holistic approach enhances our understanding of the nuanced genetic landscape of different prenatal conditions. Including a broad spectrum of cases mirrors the complexity encountered in clinical practice, making the findings particularly relevant for genetic counseling. By highlighting the distinct characteristics of isolated and non-isolated CHD and their relationships with other structural anomalies or the absence thereof, the study contributes valuable insights to guide genetic counseling practices. This comprehensive perspective is crucial for healthcare professionals in their efforts to provide informed and tailored guidance to expectant parents facing diverse prenatal conditions.

Despite its large sample size, we acknowledge the significance of addressing several limitations. First, the number of samples involving CHD is relatively small, especially for non-isolated CHD. Second, the reliance on ultrasound as an explorative technique has inherent limitations, as discerning cases with mild neurodevelopmental or craniofacial abnormalities and categorizing them into specific groups may pose challenges. Fetal and other associated symptoms may become more apparent as gestational weeks progress. Third, the study lacked parental validation and no follow-up with postnatal ultrasound information or pathological autopsy data for newborns.

Consequently, reaffirming prenatal diagnostic outcomes was unfeasible, potentially leading to the existence of false positives. Last, fetuses with Variants of Uncertain Significance did not undergo further testing, such as next-generation sequencing. These limitations should be addressed to enhance the provision of information for clinical interventions.

## Conclusion

In conclusion, applying SNP-array technology has proven highly effective in enhancing the diagnostic accuracy of abnormal copy number variations (CNVs) in fetuses with congenital heart disease (CHD). It underscores the significance of CNVs as pivotal pathogenic contributors to CHD and aids in elucidating the chromosomal etiology of affected children, thereby providing valuable guidance for families in reproductive decision-making. The comprehensive analysis reveals genetic variations in isolated and non-isolated CHD cases, encompassing common microdeletion/microduplication syndromes, chromosomal syndromes, and other diverse anomalies. The diverse genetic variations observed underscore the complexity of CHD etiology and emphasize the need for a nuanced understanding of the phenotypic expression of various chromosomal abnormality syndromes. A significant implication of the study is the recognition of the challenges inherent in relying solely on prenatal ultrasound diagnosis to distinguish between isolated and non-isolated cases of CHD. It calls for further research beyond morphological assessments, underlining the need to explore the underlying mechanisms driving congenital heart defects. Future investigations should encompass in-depth phenotypic observations integrated with comprehensive molecular genetics, metabolic studies, epigenetics, and other sophisticated analyses. Such multidimensional approaches will contribute to a more holistic understanding of CHD pathogenesis and pave the way for improved diagnostic precision and therapeutic interventions in congenital heart diseases.

### Electronic supplementary material

Below is the link to the electronic supplementary material.

Supplementary Material 1

Supplementary Material 2

Supplementary Material 3

## Data Availability

The datasets used and/or analyzed during the current study are available from the corresponding author upon reasonable request.
