# Supplementary material for: The yield of SNP microarray analysis for fetal ultrasound cardiac abnormalities
Source: BMC Pregnancy Childbirth. 2024 Apr 5;24:244. doi: 10.1186/s12884-024-06428-9 (PMC10998306; doi:10.1186/s12884-024-06428-9)
Supplement: Supplementary file 1 — Supplementary Material 1 [file 12884_2024_6428_MOESM1_ESM.docx]

Supplementary Table 1. The proportion of pathogenic findings for fetuses with congenital heart disease

| CHD classification | Isolated-CHD | | |  | Non-isolated CHD | | |
| --- | --- | --- | --- | --- | --- | --- | --- |
|  | N (%) | aneuploidy | Pathogenic CNVs |  | N (%) | aneuploidy | Pathogenic CNVs |
| **Septal defects** | 79 (8.9) | 2 | 5 |  | 19 (47.4) | 4 | 5 |
| VSD | 77 (9.1) | 2 | 5 |  | 19 (47.4) | 4 | 5 |
| ASD | 2 (0) | 0 | 0 |  | 0 (0) | 0 | 0 |
| **Conotruncal defects** | 22 (22.7) | 0 | 5 |  | 5 (60.0) | 2 | 1 |
| Tetralogy of Fallot | 10 (20.0) | 0 | 2 |  | 1 (100.0) | 0 | 1 |
| d-TGA | 7 (0) | 0 | 0 |  | 1 (100.0) | 1 | 0 |
| Interrupted aortic arch, type B | 2 (0) | 0 | 0 |  | 0 (0) | 0 | 0 |
| DORV | 3 (100.0) | 0 | 3 |  | 2 (50.0) | 1 | 0 |
| Truncus arteriosus | 0 (0) | 0 | 0 |  | 1 (0) | 0 | 0 |
| **LVOTO** | 11 (18.2) | 1 | 1 |  | 10 (30.0) | 1 | 2 |
| Coarctation of aorta | 4 (25.0) | 0 | 1 |  | 5 (0) | 0 | 0 |
| HLHS | 3 (33.3) | 1 | 0 |  | 3 (66. 7) | 0 | 2 |
| Aortic stenosis | 3 (0) | 0 | 0 |  | 1 (100.0) | 1 | 0 |
| Aortic stenosis+Pulmonary stenosis | 1 (0) | 0 | 0 |  | 1 (0) | 0 | 0 |
| **RVOTO** | 11 (0) | 0 | 0 |  | 10 (10.0) | 1 | 0 |
| Pulmonary stenosis | 11 (0) | 0 | 0 |  | 8 (12.5) | 1 | 0 |
| Pulmonary atresia | 0 (0) | 0 | 0 |  | 2 (0) | 0 | 0 |
| **AVSD** | 6 (50.0) | 2 | 1 |  | 4(100.0) | 4 | 0 |
| **Heterotaxy** | 0 (0) | 0 | 0 |  | 3 (33.3) | 0 | 1 |
| **Complex CHD** | 6 (16.7) | 1 | 0 |  | 7 (57.1) | 4 | 0 |
| Multiple, complex heart anomaly | 5 (20.0) | 1 | 0 |  | 6 (66.7) | 4 | 0 |
| Single ventricle | 1 (0) | 0 | 0 |  | 1 (0) | 0 | 0 |
| **Other CHD** | 35 (5.7) | 0 | 2 |  | 10 (40.0) | 3 | 1 |
| Right aortic arch | 11 (0) | 0 | 0 |  | 3 (33.33) | 0 | 1 |
| Persistent left superior vena cava | 10 (10.0) | 0 | 1 |  | 4 (25.0) | 1 | 0 |
| Arrhythmia | 5 (0) | 0 | 0 |  | 0 (0) | 0 | 0 |
| Aberrant right subclavian artery | 4 (0) | 0 | 0 |  | 3 (66.7) | 2 | 0 |
| Double aortic arch | 2 (50.0) | 0 | 1 |  | 0 (0) | 0 | 0 |
| Rhabdomyoma | 2 (0) | 0 | 0 |  | 0 (0) | 0 | 0 |
| Aortopulmonary window | 1 (0) | 0 | 0 |  | 0 (0) | 0 | 0 |
| **Total** | 170(11.8) | 6 | 14 |  | 68(42.6) | 19 | 10 |

*ASD*: atrial septal defect, *AVSD:* atrioventricular septal defect, *d-TGA*: dextro-transposition of the great arteries, *DORV*: double outlet right ventricle, *HLHS:* hypoplastic left heart syndrome, *LVOTO*: left ventricular outflow tract obstruction, *RVOTO*: right ventricular outflow tract obstruction, *VSD*: ventricular septal defect.
