# Supplementary material for: The yield of SNP microarray analysis for fetal ultrasound cardiac abnormalities
Source: BMC Pregnancy Childbirth. 2024 Apr 5;24:244. doi: 10.1186/s12884-024-06428-9 (PMC10998306; doi:10.1186/s12884-024-06428-9)
Supplement: Supplementary file 2 — Supplementary Material 2 [file 12884_2024_6428_MOESM2_ESM.docx]

Supplementary Table 2. Proportion of pathogenic findings for fetuses with non-isolated congenital heart disease and non-congenital heart disease combined with soft markers (%)

|  | Non-isolated CHD | | | |  | Non-CHD | | | |
| --- | --- | --- | --- | --- | --- | --- | --- | --- | --- |
|  | N | aneuploidy | Pathogenic CNVs | Likely  pathogenic CNVs |  | N | aneuploidy | Pathogenic CNVs | Likely pathogenic CNVs |
| **With single soft markers** | 39 | 13(33.3) | 4(10.3) | 0(0) |  | 370 | 34 (9.2) | 22(5.9) | 1(0.3) |
| Absent or shortened nasal bone | 4 | 0(0) | 0(0) | 0(0) |  | 61 | 1(1.6) | 4(6.6) | 0(0) |
| Choroid plexus cysts | 10 | 8(0.8) | 1(0.1) | 0(0) |  | 27 | 1(3.7) | 0(0) | 0(0) |
| Echogenic bowel | 4 | 1(25.0) | 0(0) | 0(0) |  | 1 | 0(0) | 0(0) | 0(0) |
| Echogenic cardiac focus | 0 | 0(0) | 0(0) | 0(0) |  | 15 | 1(6.7) | 2(13.3) | 1(6.7) |
| Enlarged cistern magna | 1 | 0(0) | 0(0) | 0(0) |  | 0 | 0(0) | 0(0) | 0(0) |
| Increased nuchal translucency | 3 | 0(0) | 3(100.0) | 0(0) |  | 214 | 25(11.7) | 12(5.6) | 0(0) |
| lymphatic hygroma | 1 | 1(100.0) | 0(0) | 0(0) |  | 12 | 6(54.5) | 2(18.2) | 0(0) |
| Increased nuchal fold | 0 | 0(0) | 0(0) | 0(0) |  | 2 | 0(0) | 0(0) | 0(0) |
| Mild ventriculomegaly | 3 | 0(0) | 0(0) | 0(0) |  | 25 | 0(0) | 1(4.0) | 0(0) |
| Pyelectasis | 4 | 1(25.0) | 0(0) | 0(0) |  | 7 | 0(0) | 0(0) | 0(0) |
| Persistent right umbilical vein | 1 | 0(0) | 0(0) | 0(0) |  | 4 | 0(0) | 0(0) | 0(0) |
| Shortened long bones | 1 | 0(0) | 0(0) | 0(0) |  | 0 | 0(0) | 0(0) | 0(0) |
| Single umbilical artery | 7 | 2(28.6) | 0(0) | 0(0) |  | 2 | 0(0) | 1(50.0) | 0(0) |
| **With multiple soft markers** | 13 | 4(30.8) | 4(30.8) | 0(0) |  | 45 | 7(15.6) | 0(0) | 0(0) |
| **Without soft markers** | 16 | 2(12.5) | 2(12.5) | 0(0) |  | 123 | 5(4.1) | 8(6.5) | 1(0.8) |
| **Total** | 68 | 19(27.9) | 10(14.7) | 0(0) |  | 538 | 46(8.6) | 30(5.6) | 2(0.4) |
