# Supplementary material for: The yield of SNP microarray analysis for fetal ultrasound cardiac abnormalities
Source: BMC Pregnancy Childbirth. 2024 Apr 5;24:244. doi: 10.1186/s12884-024-06428-9 (PMC10998306; doi:10.1186/s12884-024-06428-9)
Supplement: Supplementary file 3 — Supplementary Material 3 [file 12884_2024_6428_MOESM3_ESM.docx]

Supplementary Table 3. The proportion of pathogenic findings for control group (%)

|  | N | Aneuploidy | Pathogenic CNVs | Likely pathogenic CNVs |
| --- | --- | --- | --- | --- |
| advanced paternal age | 488 | 15(3.1) | 7(1.4) | 2(0.4) |
| NIPT positive results | 241 | 41(17.0) | 23(9.5) | 1(0.4) |
| High risk of maternal serum screening | 412 | 22(5.3) | 7(1.7) | 0(0) |
| adverse pregnancy history | 151 | 0(0) | 4(2.6) | 0(0) |
| Others | 24 | 0(0) | 1(4.2) | 0 |
| Total | 1316 | 78(5.9) | 42(3.2) | 3(0.2) |
